# Does Innovation Efficiency Suppress the Ecological Footprint? Empirical Evidence from 280 Chinese Cities

**DOI:** 10.3390/ijerph17186826

**Published:** 2020-09-18

**Authors:** Haiqian Ke, Wenyi Yang, Xiaoyang Liu, Fei Fan

**Affiliations:** 1 Institute of Central China Development, Wuhan University, Wuhan 430072, China; kehaiqian@whu.edu.cn; 2 School of Economics and Management, Nanyang Institute of Technology, Nanyang 473000, China; 3 Institute of Regional and Urban-Rural Development, Wuhan University, Wuhan 430072, China; wenyiyang@whu.edu.cn; 4 School of Architecture, Tianjin University, Tianjin 300072, China

**Keywords:** innovation efficiency, ecological footprint, threshold regression, mediating effect

## Abstract

Innovation is an important motivating force for regional sustainable development. This study measures the innovation efficiency of 280 cities in China from 2014–2018 using the super-efficiency slack-based measure and it also analyzes its impact on the ecological footprint using the generalized spatial two-stage least squares (GS2SLS) method and uses the threshold regression model to explore the threshold effect of innovation efficiency on the ecological footprint at different economic development levels. We find the corresponding transmission mechanism by using a mediating effect model. The major findings are as follows. First, we find an inverse U-shaped relationship between innovation efficiency and the ecological footprint for cities across China as well as in the eastern and central regions. That is, innovation efficiency promotes then suppresses the ecological footprint. Conversely, in western and northeastern China, improvements in innovation efficiency still raise the ecological footprint. Second, for the entire country, as economic development increases from below one threshold value (4.4928) to above another (4.8245), the elasticity coefficient of innovation efficiency to the ecological footprint changes from −0.0067 to −0.0313. This indicates that the ability of innovation efficiency improvements to reduce the ecological footprint is gradually enhanced with increased economic development. Finally, the industrial structure, the energy structure, and energy efficiency mediate the impacts of innovation efficiency on the ecological footprint.

## 1. Introduction

Despite significant global development in industrialization and urbanization, demand for Earth’s resources has exceeded reasonable limits. Global warming, environmental pollution, and serious resource depletion have caused severe problems worldwide. The current ecological and environmental crisis threatens the sustainable development of humans and the regional environment [1]. A continuous increase in the global ecological footprint caused by economic growth, industrial structure changes, and increased energy consumption was revealed by measuring the ecological footprint of 144 countries from 1988 to 2008 [2]. China’s Ecological Footprint Report 2015, published by the World Wide Fund for Nature [3], reported that China is consuming at a rate of 2.2× its ecological resources. Indeed, China’s ecological footprint now accounts for one-sixth of the world’s total, more than any other country. Thus, considerable research should be conducted into China’s ecological footprint.

The ecological footprint concept was first proposed by the Canadian ecological economist Rees in 1992 [4]. It describes the area of biologically productive land required by humans to produce the necessary resources and absorb the generated waste under certain demographic and economic conditions. In other words, it reflects the impact of human activities on the natural ecological environment. The ecological footprint not only reflects individual or regional resource consumption intensity but can also objectively measure and compare temporal and spatial sustainability. Therefore, it can measure human-induced stress to the ecological environment [5]; the larger the footprint, the more serious is the damage to the ecological environment [6].

Previous studies have suggested that the impact of technological innovation on the ecological environment exhibits complex “duality” [7]. On the one hand, improvements in technological innovation can improve energy efficiency (e.g., by replacing fossil fuels with clean energy) and CO_2_ emission treatment technology, thereby improving the efficiency of natural resource utilization, reducing environmental pollution caused by carbon emissions, and contributing to the “green” development of the ecological environment [8]. Similar studies have shown that increasing technological innovation has significantly reduced the deterioration of the ecological environment in various provinces in China [9]. However, as in developed countries, China’s ecological problems are largely due to industrialization [10]. Technological innovation promotes production efficiency and accelerates the large-scale expansion of industry, bringing economic benefits but also increasing the excessive consumption of resources [11]. Therefore, industrialized regions are impacted by resource depletion and the deterioration of the ecological environment. In addition, although technological innovation can improve energy efficiency, it only slightly reduces energy consumption, as it is impossible to reduce most of the energy use. For example, if energy prices fall because of improved energy efficiency, lower prices may encourage humans to use more energy, which in turn places greater pressure on the ecological environment [12]. Therefore, research has not yet reached a clear conclusion on the relationship between technological innovation and the ecological environment.

Innovation efficiency refers to the allocation and utilization efficiency of scientific and technological resources over space and time [13], which reflects the strength of regional technological innovation capabilities [14]. The ecological footprint, as a comprehensive indicator reflecting the quality of the ecological environment, can measure human pressure on the environment. Therefore, it is important to evaluate the impact of innovation efficiency on the ecological footprint, the relationship between the two measures, and the effect of regional economic development on this relationship. Although studies have investigated many of the factors influencing the ecological footprint [15], few have analyzed the mechanism of these impacts from the perspective of innovation efficiency. In addition, as the ecological footprint is not simply a local ecological problem, China’s per capita ecological footprint is not completely random and exhibits significant spatial agglomeration [16]. Therefore, when discussing the impact of innovation efficiency on the ecological footprint, it is necessary to consider the spatial correlation of the ecological footprint itself to obtain more accurate research results.

Based on the foregoing, this study uses the super-efficiency slack-based measure (SBM) model to calculate the innovation efficiency of 280 cities in China from 2014–2018 and then conducts an empirical analysis of the impact of innovation efficiency on the ecological footprint using the generalized spatial two-stage least squares (GS2SLS) model, which can control for both the spatial effect and the endogenous effect. This study thus contributes to environmental science and public health research in the following three aspects. First, the impact of urban innovation efficiency on the ecological footprint is systematically analyzed. Considering that the core spatial carriers of the national innovation system are cities, 280 Chinese cities above the prefecture level are used as the research objects. Second, a threshold regression model is employed to investigate the regional and staged impact of innovation efficiency on the ecological footprint under different economic development levels. Night light data are used as a threshold variable to characterize the economic development level of a region because it can test real economic growth and measure economic agglomeration, urbanization, population mobility, energy consumption, and other economic activities [17]. Finally, following [18], this study constructs a mediating effect model composed of three regression equations to identify the transmission mechanism of innovation efficiency on the ecological footprint.

## 2. Literature Review

Research on the relationship between technological innovation and the ecological environment is predominantly conducted from three viewpoints. The first is that technological innovation can improve the ecological environment. According to the Porter hypothesis, stimulating the “innovation compensation” effect of enterprises through technological innovation is a key method of reducing pollution emissions [19,20]. The IPAT model (environmental impact (I) = population (P) × affluence (A) × technology (T)) links the environmental impact with the population size, per capita wealth, and technological level, suggesting that technological innovation and progress can alleviate the environmental pollution caused by population growth [21]. One study found that the technological innovation capabilities of 30 provinces in China have a positive effect on the structure of regional economic growth, resource utilization, and the ecological environment; simultaneously, the spillover effect of regional technological innovation also has a positive effect on the ecological environment for three main reasons [22]: (1) it improves energy efficiency and reduces energy consumption by using a more environmentally friendly combination of production methods, (2) it develops new energy sources and reduces the over-exploitation of resources, and (3) it promotes low-carbon technology and improves the efficiency of pollution control. Therefore, increased technological innovation can protect and improve the ecological environment [23,24,25].

The second viewpoint is that not all technological innovation can improve the ecological environment. The greater the technological innovation, the higher is the degree of environmental pollution in areas with more advanced industrial development. This is observed in eastern and central China, where the quality of the ecological environment is far below the national average [26]. Several studies have shown that technological innovation has a significant destructive effect on the ecological environment, as enterprises in the initial stage of industrial agglomeration do not accumulate a large amount of human capital and there is insufficient motivation for the innovation of clean production technology in the agglomeration area. Hence, in terms of technology research, development, and implementation, greater focus is placed on how to improve the level of product technology to increase firm profits, neglecting environmental protection technology [27,28,29]. Therefore, the pollution suppression effect of technological innovation is offset or even concealed by the environmental damage effect of industrial enterprises. In addition, when industrial agglomeration is small, the infrastructure is not yet complete, public pollution control facilities have not yet been built, and resource allocation has not yet reached the optimal state, which is not conducive to reducing the marginal pollution control costs of enterprises [30,31].

The third perspective is that the impact of technological innovation on the ecological environment has an inverse U-shaped relationship. For example, in eastern and central China, the relationship between technological innovation and ecological pollution has a clear inverse U-shaped curve, whereby technological innovation first promotes then inhibits environmental pollution [32]. Technological innovation and progress can also curb carbon emissions in the long term, but not in the short term [33]. Therefore, technological innovation may exhibit a nonlinear relationship with initial destruction followed by an improvement in the ecological environment. Hence, the impact of innovation efficiency on the ecological footprint may also have an inverse U-shaped relationship characterized by inhibition then promotion. Hence, the following hypothesis is proposed:

**Hypothesis 1** **(H1).** The impact of innovation efficiency on the ecological footprint exhibits an inverse U-shaped trend.

China has a vast territory and the intensity of natural resource utilization and ecological environment structure exhibit clear spatial heterogeneity between regions. China’s ecological footprint has risen rapidly since 2000 [34], with the highest ecological footprint in the east, followed by a “stepped” spatial distribution in central and western regions. In addition, China’s regional innovation efficiency is characterized by heterogeneity and agglomeration [35]. Moreover, the innovation efficiency of provinces in China declines from east to west [36,37]. Therefore, innovation efficiency may have a heterogeneous impact on the ecological footprint of different regions; however, previous or current research has not fully resolved this issue.

In addition, as economic development is relatively high in eastern cities, technological innovation has improved the ecological environment in eastern China more significantly than that in other regions [38]. The inhibitory effect of technological innovation on China’s carbon emission reductions is positively affected by the regional economic development, as when the economic development is high, the greater financial support required for the development and application of technological innovation is available, which is conducive to vigorously developing, promoting, and utilizing clean energy, reducing carbon emissions, and suppressing pollutant emissions [39]. Further, a higher economic development leads to a greater awareness of social environmental protection as well as a gradual shift in consumers’ focus from the price of final products to environmental protection and energy conservation during the production process, which has a positive impact on the ecological environment [40]. Thus, under different levels of regional economic development, technological innovation may result in varying degrees of improvement to the ecological environment. The second hypothesis is therefore proposed:

**Hypothesis 2** **(H2).** The impact of innovation efficiency on the ecological footprint exhibits regional differences; under different economic development levels, the impact of innovation efficiency on the ecological footprint exhibits a threshold effect.

**Figure 1:**
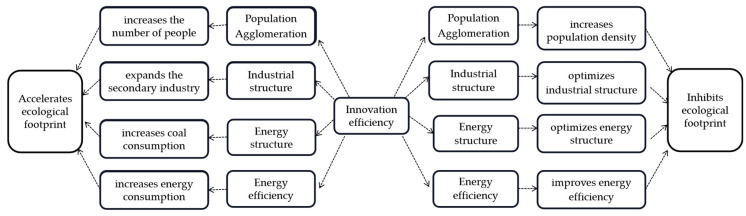
Mechanisms of the impacts of innovation efficiency on the ecological footprint.

Changes in the ecological footprint are affected by many social, economic, and natural factors such as population, consumption, land, climate, technology, and management, each with complex nonlinear characteristics. Therefore, a review of the previous literature [41], as shown in Figure 1, suggests that innovation efficiency may affect the ecological footprint in four ways: population aggregation, the industrial structure, the energy structure, and energy efficiency.

First, the improvement in urban innovation efficiency leads to a more rapid urban population agglomeration supported by R&D and service industry personnel, which affects the ecological footprint [42]. Population agglomeration refers to an increase in urban population density, which translates into shorter commuting distances, reduced car usage per capita, and fewer pollutant gas emissions [43]. In addition, population agglomeration leads to the concentration of enterprises and public facilities, which is conducive to the centralized construction of infrastructure [44]. In particular, this results in sharing environmental pollution control facilities in the centralized infrastructure, reducing the effect of pollution diffusion, and taking advantage of economies of scale and agglomeration to improve the ecological environment [45]. Therefore, an improvement in innovation efficiency may reduce the ecological footprint through population agglomeration.

Second, the industrial structure typically refers to the proportions of the primary, secondary, and tertiary industries in the economy, with the consumption of ecological resources by the primary and secondary industries serving as an important driving force for the continuous increase in the ecological footprint [46]. This is mainly because the primary and secondary industries account for a relatively higher degree of natural resources and pollution when compared to the tertiary industry. However, the process of improving innovation efficiency guides the flow of innovation resources such as research and development (R&D) funds and R&D capital to more efficient departments and then promotes a further accumulation of innovation resources. Simultaneously, this also improves the industrial technology and output quality, which continuously increase the proportion of the tertiary industry (characterized by high added value and low energy consumption) [47] and gradually reduce the proportion of the primary and secondary industries (characterized by high pollution, high energy consumption, and low added value). This in turn promotes the greater rationalization of the regional industrial structure and improves the ecological environment. Therefore, improved innovation efficiency may restrain the ecological footprint by optimizing and upgrading the industrial structure.

Third, optimizing China’s energy structure aims to gradually reduce its dependence on coal and increase the use of cleaner and more sustainable energy [48]. Keeping all other conditions constant, a 1% increase in technological innovation in China’s 30 provinces from 1997 to 2014 reduced the average proportion of coal consumption in China by 0.732% [49,50]. Therefore, increasing technological innovation can significantly reduce the consumption of traditional coal energy in the production process [51], thereby improving the ecological environment. In addition, technological innovation can promote the development of renewable energy and increase the supply capacity of renewable energy [52] to meet energy demand and optimize the energy structure [53], which again improves the ecological environment. As innovation efficiency is an important driving force for improved technological innovation, the ecological footprint is an effective indicator of the ecological environment. As such, an optimized energy structure also suppresses increases in the ecological footprint.

Fourth, during economic development or industrial production, technological innovation is an important factor affecting the energy efficiency of a region [54] because improvements in technological innovation promote clean environmental energy in the production process, thereby reducing pollutant emissions [55]. Moreover, energy savings and effective energy use increase with increasing energy efficiency, reducing excessive energy consumption and helping limit the ecological footprint. Therefore, the third hypothesis is proposed:

**Hypothesis 3** **(H3).** Innovation efficiency can affect the ecological footprint through population aggregation, the industrial structure, the energy structure, and energy efficiency.

## 3. Materials and Methods

### 3.1. Models

#### 3.1.1. STIRPAT Model

Based on the stochastic impacts by regression on population, affluence, and technology (STIRPAT) model [56] and environmental Kuznets curve hypothesis [57], the following benchmark model is first constructed to investigate the impact of innovation efficiency on the ecological footprint (Hypothesis 1):

(1) $$\begin{array}{l} lnEF_{it}=\alpha_{0}+\alpha_{1}{lnpop}_{it}+\alpha_{2}{lngdp}_{it}+\alpha_{3}{\left({lngdp}_{it}\right)}^{2}+\alpha_{4}{lntec}_{it}\\ +\alpha_{5}{lnie}_{it}+\alpha_{6}{\left({lnie}_{it}\right)}^{2}+\alpha_{7}X_{it}+\varepsilon_{it} \end{array}$$

where *i* is the cross-sectional unit of 280 prefecture-level cities in China (in 2017, there were 298 prefecture-level cities in China; 18 in the western region with missing statistical data were excluded here) and *t* represents the year. The population, P, GDP, A, and technology level, T, are respectively characterized by *lnpop_it_*, *lngdp_it_*, and *lntec_it_*. *lnEF_it_* is the ecological footprint of the dependent variable, *lnie_it_* is the core dependent variable of innovation efficiency, *X_it_* is a set of control variables, *α0–α6* and *α7* are the parameters to be estimated, and ε is the random disturbance term.

#### 3.1.2. Threshold Regression Model

A threshold regression model is constructed based on Hypothesis 2 to verify whether regional innovation efficiency has different effects on the ecological footprint under heterogeneous economic development levels [58]. First, the traditional single-threshold regression model is set:

(2) $$lnEF_{it}=\alpha X_{it}+\beta_{1}{lnie}_{it}\times I\left(T_{it}\leq \delta \right)+\beta_{2}{lnie}_{it}\times I(T_{it}>\delta )+C+\varepsilon_{it}$$

where *lnEF_it_* is the explained variable of the *i*-*th* region in year *t*, *X* is the control variable, *lnie_it_* is the core explanatory variable, and *T* is the threshold variable (i.e., economic development; represented by the night light data). *δ* is the fixed threshold, *α* is the influence coefficient of *lnie_it_* on the explained variable, *β*_1_ and *β*_2_ are the influence coefficients of the core explanatory variable *lnie_it_* on the explained variable when *Tit* ≤ *δ* and *Tit* > *δ*, respectively, *C* is a constant term, *ε_it_*~(0, *σ*) is a random disturbance term, and *I* is an indicator function. The value of *I* (i.e., economic development) depends on whether the conditions in parentheses are established. When the corresponding conditions are established, the value is 1; otherwise, the value is 0.

Equation (2) only assumes one threshold, but two or more thresholds may exist in reality. To make the analysis more accurate, we set a double-threshold model and a triple-threshold model. Equations (3) and (4) show the equations for the double-threshold and triple-threshold tests; the meanings of *β*_2_ and *β*_3_ are similar to those of *β*_1_. Thresholds above the triple threshold are not discussed in this study.

(3) $$lnEF_{it}=\alpha X_{it}+\beta_{1}{lnie}_{it}\times I\left(T_{it}\leq \delta_{1}\right)+\beta_{2}{lnie}_{it}\times I(\delta_{1}<T_{it}\;\leq \delta_{2})+\beta_{3}{lnie}_{it}\times I(T_{it}>\delta_{2})+C+\varepsilon_{it}$$

(4) $$\begin{array}{l} lnEF_{it}=\alpha X_{it}+\beta_{1}{lnie}_{it}\times I\left(T_{it}\leq \delta_{1}\right)+\beta_{2}{lnie}_{it}\times I(\delta_{1}<T_{it}\leq \delta_{2})\\ +\beta_{3}{lnie}_{it}\times I(\delta_{2}<T_{it}\leq \delta_{3})+\beta_{4}{lnie}_{it}\times I(T_{it}>\delta_{3})+C+\varepsilon_{it} \end{array}$$

#### 3.1.3. Mediating Effect Model

According to Hypothesis 3, innovation efficiency affects the ecological footprint through four methods: population aggregation, the industrial structure, the energy structure, and energy efficiency. A mediating effect model composed of the following three regression equations is constructed to identify and test the above mechanisms:

(5) $$lnEF=\theta_{0}+\theta_{1}{lnie}_{it}+\theta_{2}ln{\left({ie}_{it}\right)}^{2}+\theta_{3}Y_{it}+\zeta_{it}$$

(6) $$D_{it}=\beta_{0}+\beta_{1}{lnie}_{it}+\beta_{2}ln{\left({ie}_{it}\right)}^{2}+\beta_{3}Y_{it}+\mu_{it}$$

(7) $$lnEF_{it}=\gamma_{0}+\gamma_{1}{lnie}_{it}+\gamma_{2}ln{\left(ie_{it}\right)}^{2}++\gamma_{3}Y_{it}+\gamma_{4}D_{it}+\tau_{it}$$

where *Y_it_* is a vector set composed of the control variables; *D_it_* is a possible mediating variable, including population aggregation (*lnmidu*), the industrial structure (*2ndchange*), the energy structure (*lngas*), and energy efficiency (*lneefcy*); and *lnie_it_* and *lnEF_it_* are innovation efficiency and the ecological footprint, respectively. According to the principle of the mediation effect model, a mediating effect is indicated if the coefficients *θ_1_* or *θ_2_*, *β_1_* or *β_2_*, or γ_4_ are significant and the coefficients *γ*_1_ and *γ*_2_ are smaller than *θ_1_* and *θ_2_* or the degree of significance decreases.

### 3.2. Variable Selection and Description

#### 3.2.1. Dependent Variable (Ecological Footprint)

According to the definition of the ecological footprint, we divide usable land into six types of land for ecological production and the absorption of waste: cultivated land, fossil energy land, grassland, water area, forest land, and construction land. We then multiply these land types by the corresponding equilibrium factors. The main function of the ecological footprint is to convert multiple complex natural resources into the same coordinate system for the calculation. Any differences in equilibrium factors between years given by different institutions and researchers are small and relatively stable. This study uses the 2018 equilibrium factor data provided by the Global Footprint Network [59]: cultivated land = 2.52, grassland = 0.43, forest land = 1.28, water area = 0.35, fossil energy land = 1.28, and construction land = 2.52.

(8) EF=N×ef

(9) $$ef=\sum_{j=1}^{6}\sum_{i=1}^{n}(r_{j}a_{i})=\sum_{j=1}^{6}\sum_{i=1}^{n}(r_{j}\times c_{i}/p_{i})\;j=\left(1,\;2,\;3,\;\ldots \;6\right)$$

**Figure 2:**
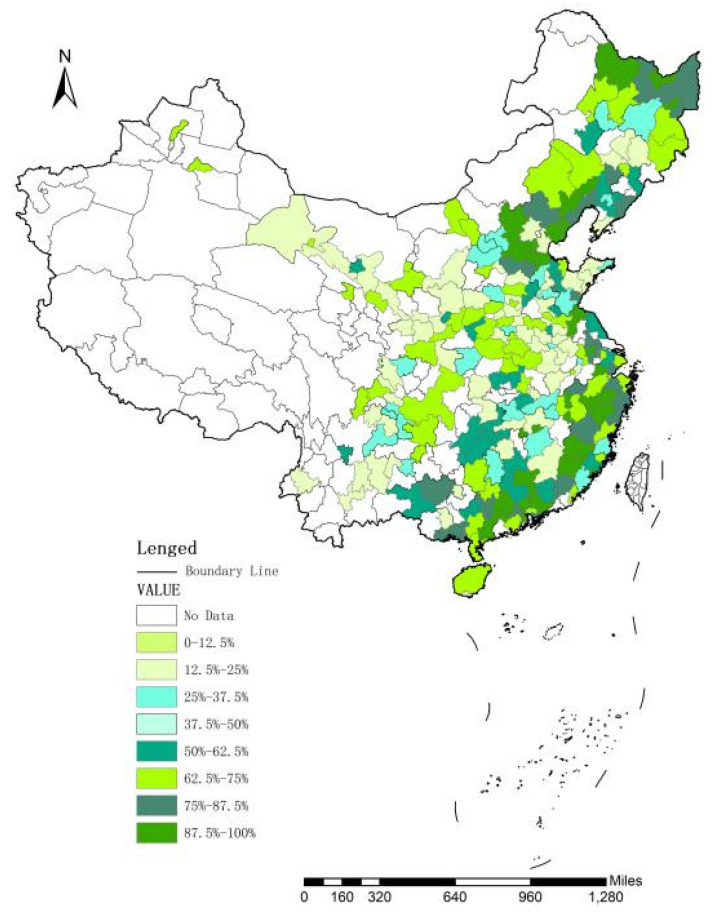
China’s urban ecological footprint in 2014.

**Figure 3:**
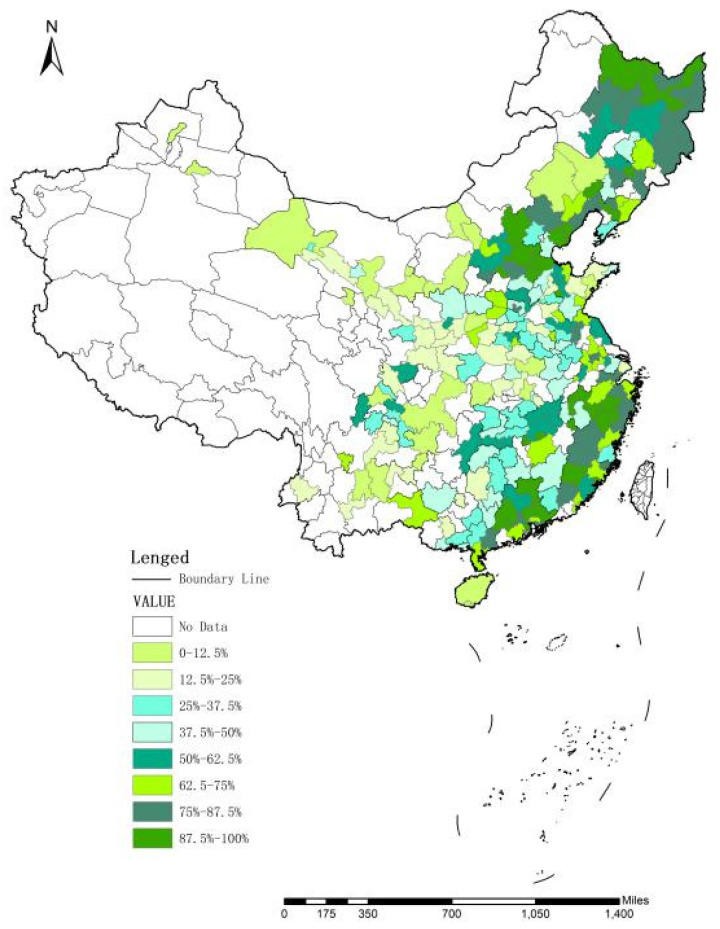
China’s urban ecological footprint in 2018.

In Equation (8), *EF* is the ecological footprint of the region, *ef* is the ecological footprint per capita of the region, and *N* is the regional population. In Equation (9), *i* is the category of consumption resources, *a_i_* is the ecological productive land per capita, converted according to the average output of the *i-th* consumption resource in the world, *c_i_* is the per capita production of the *i-th* consumer resource, *p_i_* is the global average output of the *i-th* consumption resource produced by the ecologically productive land, and *rj* is the equilibrium factor of the *j-th* ecologically productive land. There are six types of ecologically productive land and the ecological footprint of the city is calculated based on the above equilibrium factors. This study uses the arc geographic information system (ArcGIS) natural fracture method to divide the ecological footprint of Chinese cities into eight levels. Figure 2 and Figure 3 show the distribution of the ecological footprint of 280 cities across China in 2014 and 2018, respectively. The darker the color, the greater are the ecological footprint and the deterioration of the ecological environment.

First, a comparison of Figure 2 and Figure 3 indicate that the ecological footprint in China has deteriorated from 2014 to 2018; and, if we divide China into four parts (with the eastern region including Beijing, Tianjin, Hebei, Shanghai, Jiangsu, Zhejiang, Fujian, Shandong, Guangdong and Hainan; the central region including Shanxi, Anhui, Jiangxi, Henan, Hubei and Hunan; the western region including Inner Mongolia, Guangxi, Chongqing, Sichuan, Guizhou, Yunnan, Shaanxi, Gansu, Qinghai, Ningxia and Xinjiang; and the northeast region including Liaoning, Jilin and Heilongjiang), the rate of deterioration has decreased and the ecological footprint tended to shift from the east to central and western regions. Second, the average ecological footprint of cities is not uniformly distributed nationally; rather, there is a clear spatial agglomeration and relevance characterized by the decreasing pattern from eastern to central to western China. Therefore, the GS2SLS model, which can control for spatial spillovers, is used to explore the impact of China’s urban innovation efficiency on the ecological footprint.

#### 3.2.2. Core Independent Variable (Innovation Efficiency)

This study employs the SBM model to measure China’s urban innovation efficiency under environmental constraints from 2014 to 2018 because traditional data envelopment analysis does not consider the effect of slack variables, random error terms, or the external environment [60]. To address the limitations of traditional data envelopment analysis, a super-efficiency SBM was proposed based on previous studies [61]. The improved model solves the problems of nonzero slack between the inputs and outputs and undesired outputs in the production process as well as distinguishes the efficiency of decision-making units.

In this study, we measure regional innovation efficiency from the perspective of the inputs and outputs of scientific and technological resources. Regional innovation efficiency capabilities are mainly reflected in the allocation of scientific and technological human resources, financial resources, and information resources [62]. Therefore, these aspects are considered as the research objects of innovation efficiency. The full-time equivalent of R&D personnel, which reflects the ability to attract regional talent, is used to represent the human resources of science and technology. The financial resources of science and technology are represented by internal R&D expenditure, which reflects the regional support for regional science and technology activities. Finally, the number of Internet users reflects the development of regional science and technology information resources. In addition, knowledge innovation outputs and technological innovation outputs are the expected outputs of scientific and technological resources. The number of scientific papers represents the level of knowledge innovation and the number of patent applications represents the level of technological innovation. The number of patent authorizations is more uncertain than the number of patent applications due to the influence of human factors [63] (e.g., patent authorization agencies). Therefore, the number of patent applications is a more suitable measure of the true level of scientific and technological resource outputs.

#### 3.2.3. Control Variables

Changes in the ecological footprint are affected by many factors such as the economy, society, environment, and technology [64]. This study employs the STIRPAT model [65] and selects the following six control variables: population (units of 10,000 people), GDP per capita (units of 10,000 Yuan per person), technical level (units of %), household consumption (units of 10,000 Yuan), proportion of pollution control investment to GDP (units of %), and proportion of the secondary industry (units of %). Among them, population size, GDP per capita [66], household consumption [67], and proportion of the secondary industry [68] are expected to have a positive effect on the ecological footprint. Conversely, the technological level [69] and proportion of pollution control investment to GDP are expected to have a negative effect [70]. In addition, Stata 15 software indicates that the variance inflation factors (VIFs) of all the variables are less than four and that the average VIF value is 2.66 (<10); that is, the collinearity test is passed.

#### 3.2.4. Threshold Variable (Night Light Data)

This study employs stable lighting data from 2014 to 2018 for 280 cities in China to measure regional economic development. The Defense Meteorological Program (DMSP) Operational Line-Scan System data are widely used to estimate the population size/electricity consumption and monitor urban expansion. First, night light data are a good data source for research monitoring human activities. Second, stable lighting data can be used not only to test real economic growth but also to measure economic activities such as economic agglomeration, urbanization, population mobility, and energy consumption [71]. Third, night light data objectively reflect regional differences in the production and living conditions of the society [72]. Therefore, night light data are an objective measure of urban economic development. However, because the data values are relatively large (i.e., in the whole data range, the sensitivity to differences is greater for lower values than higher values), the logarithms of these values are used instead. This approach compresses the scale of the variables and reduces the absolute value of the data but does not change the nature of the data or the correlation. Additionally, the logarithms are easy to calculate and apply to the models.

#### 3.2.5. Mediating Variables

We select four variables to characterize population aggregation, the industrial structure, the energy structure, and energy efficiency. First, the proportion of population in the administrative area is used to measure the population accumulation effect. The greater the population density, the greater are the concentration of infrastructure construction, sharing of environmental pollution treatment facilities, and reduction in pollution diffusion effects, which may suppress the ecological footprint. That is, the greater the population density, the smaller is the ecological footprint; therefore, the proportion is expected to be negative. Second, the proportion of the added value of the secondary industry to GDP is used to characterize the change in the industrial structure. This coefficient is expected to be positive because the secondary industry has higher fossil energy consumption and pollution emissions [73], which may increase the ecological footprint. Third, the proportion of annual coal consumption to total energy consumption is used to characterize the energy structure effect. China’s main energy structure is dominated by coal, which is also the main source of China’s environmental pollution problems [74]. More coal consumption results in greater environmental pollution, which may increase the ecological footprint. Therefore, the coefficient is expected to be positive. Finally, the proportion of energy consumption to GDP is used to characterize energy efficiency. The lower the energy consumption per 10,000 Yuan of GDP, the higher is the energy efficiency. By saving energy and improving pollution control technology [75], pressure on the ecological environment decreased and the ecological footprint is reduced. Therefore, the impact of energy consumption to GDP on the ecological footprint is expected to be positive. According to Stata 15 software, the VIF values of the four variables are all less than 2 and the average VIF value is 1.38 (<10), indicating that the collinearity test is passed. Table 1 shows the variables.

### 3.3. Spatial Weight Matrix

China’s natural resource endowment and socioeconomic development vary greatly by region and exhibit a strong spatial correlation with the ecological footprint [76]. Therefore, ecological footprint research should include a weight matrix that reflects this spatial relationship in the model. Based on the geographical distance between cities, this study constructs a geographical distance spatial weight matrix (W_1_) to reflect the influence of geographical factors on the spatial distribution characteristics of the ecological footprint. Among them, w*_ij_* of W_1_ represents the nearest highway (in miles) between city *i* and city *j*. In addition, the economic geographical matrix W_2_, which simultaneously reflects the city’s economic and geographical information, is obtained through matrix & laboratory (MATLAB) dot multiplication and used to test the robustness of the results [77], where W_2_ = ωW_1_ + (1-ω)W_3_, ω denotes the weight of the geographical distance spatial weight matrix (0.5), W_3_ represents the economic distance spatial weight matrix, and *w_ij_* is the reciprocal of the absolute difference between the annual average GDP per capita of city *i* and city *j*.

### 3.4. Endogeneity Problems

Two-way causality between the explanatory variable and explained variable may lead to the existence of endogeneity problems. An improvement in innovation efficiency can promote industrial upgrading and increase energy efficiency and pollution treatment efficiency, thereby improving the ecological environment and reducing the ecological footprint. In turn, the Porter hypothesis proposes that the consequence of imposing environmental regulations on enterprises is higher environmental standards, which can promote continuous technological innovation [78], thereby improving innovation efficiency. Severe endogeneity problems can make the ordinary least squares method biased and inconsistent, and the maximum likelihood estimation method will also fail when there is a heteroscedasticity problem. It is possible to select the lag term of the explanatory variable as an instrumental variable to solve the problem of an invalid estimation (i.e., use the Two-Stage Least Squares (2SLS) method for the estimation). However, considering the spatial spillover effect of the ecological footprint [79], the GS2SLS estimation is instead used to select the explanatory variables and their spatial lags as instrumental variables. We then estimate the spatial panel model using the 2SLS method, while controlling for the spatial correlation effects and endogeneity problems in the model [80]. In the benchmark regression, the highest third-order spatial lag is selected as the instrumental variable. (The highest second-order spatial lag is selected as the instrumental variable in the robustness test.)

## 4. Results and Discussion

### 4.1. Benchmark Regression Analysis

Table 2 shows the GS2SLS estimation results of the benchmark model. Columns (1) and (2) are the estimation results of the fixed effects model and random effects model that only consider the core explanatory variables and basic variables of the STIRPAT model, respectively. Columns (3) and (4) add the other control variables. The Hausman test in Columns (1)–(4) passes the 1% significance level, indicating that the fixed effects model should be selected.

The coefficients of the spatial lag of the ecological footprint (*w*_1_*lnEF) in Table 2 are all significantly positive at the 1% level, indicating that the ecological footprint has a positive and significant spatial spillover effect. In other words, areas with higher ecological footprints raise the ecological footprint of neighboring areas. This is because the significant differences in the abundance of natural resources in different cities in China lead to a diffusion-style flow of natural resources to nearby regions [81]. Moreover, human factors such as industrial transfer, cross-regional trade, and environmental policy externalities further strengthen the spatial correlation between regional innovation efficiency and the ecological footprint [82]. The regression results in Columns (1) and (3) show that the first coefficient of the core explanatory variable of innovation efficiency (*lnie*) is significantly positive and the quadratic coefficient of *ln(ie)*^2^ is significantly negative, indicating a significant inverse U-shaped relationship between innovation efficiency and the ecological footprint. Therefore, Hypothesis 1 is confirmed. An improvement in innovation efficiency first promotes then inhibits the ecological footprint. This may be because the ecological footprint is not only directly affected by economic factors such as economic scale, the industrial structure, technological progress, and international trade, but also indirectly affected by environmental regulation, citizens’ environmental awareness, and environmental education [83]. Therefore, the impact of innovation efficiency on the ecological footprint may be influenced by other factors, forming a complex relationship. In addition, the initial stages of innovation efficiency improvement do not reduce industrial pollution in the short term, but may drive enterprises to larger-scale production and resource utilization. However, as no positive externalities of new technology use on pollution have been discovered, the degree of pollution to the ecological environment will increase, raising the ecological footprint. Conversely, long-term improvement in innovation efficiency results in a higher input/output ratio of scientific and technological resources, which has an important role in increasing technological innovation, improving energy efficiency, and reducing energy consumption through a more environmentally friendly combination of production methods. This in turn allows the development of new energy sources to reduce the excessive exploitation and use of resources, promotes low-carbon technology, and improves pollution control efficiency. These factors are crucial for protecting the ecological environment and limiting the ecological footprint [84,85].

As shown in Columns (1) and (3) in Table 2, population (*lnpop*) plays a significant role in raising the ecological footprint. The coefficient of per capita GDP (*lngdp*) is significantly positive and the quadratic coefficient is significantly negative, indicating that the traditional environmental Kuznets curve hypothesis between economic growth and the ecological footprint is satisfied (i.e., there is an inverse U-shaped relationship); this conclusion is consistent with those of other studies that have reported that improvements in China’s economic development will eventually significantly inhibit growth in the ecological footprint [86,87]. China is in a critical period of transition from extensive to intensive economic growth [88], with innovation the essential driving force behind development, accelerated transformation of the development mode, optimized economic structure, and transformation of growth momentum. Therefore, improving innovation efficiency and accelerating the transformation and application of scientific and technological achievements are crucial for reducing the ecological footprint. Technological level (*lntec*) has a significant inhibitory effect on the ecological footprint, mainly because technological progress can improve energy use and pollution control efficiency, thereby raising the quality of the ecological environment and reducing the ecological footprint. Although the relationship between increasing consumption (*lncosmp*) and the ecological footprint is positive, it is not significant; therefore, the impact on the ecological footprint is still unclear. The higher the pollution control investment (*lnpolut*), the higher is the quality of the ecological environment and the more significant is the suppression of the ecological footprint. Finally, although higher output in the secondary industry (*lnsec*) leads to greater consumption of natural resources and greater pressure on the ecological environment, the coefficient is small and not significant, indicating that the proportion of the added value of the secondary industry to GDP does not influence the ecological footprint. Furthermore, this indicates that China’s industrial structure is becoming more optimized and economic growth mode is becoming increasingly environmentally friendly.

### 4.2. Robustness Test

To test the robustness of the benchmark regression results, this study adopts three main methods: replacing the spatial weight matrix, replacing the instrumental variable, and adjusting the weight coefficient. First, we select the geographical and economic distance nested weight matrix (W_2_) to replace the geographical distance spatial weight matrix (W_1_) used in the previous regression. W_2_ not only considers the role of geographical factors but also reflects the fact that economic factors have spatial relevance, which can more comprehensively reflect the spatial relevance of the research object. Second, based on the GS2SLS regression, the highest second-order spatial lag is used for the re-estimation, replacing the highest third-order spatial lag used in the previous regression as an instrumental variable. Third, we set the weight of the geographical distance spatial weight matrix to 0.7 and re-estimate Equation (1). The ecological footprint spatial lag in Columns (1)–(3) in Table 3 is still significantly positive and an inverse U-shaped relationship remains between the core explanatory variable (innovation efficiency) and ecological footprint. The benchmark regression results thus have strong robustness.

### 4.3. Regional Differences in the Impact of Innovation Efficiency on the Ecological Footprint

Table 4 reports the GS2SLS estimation results of the impact of innovation efficiency on the ecological footprint in the eastern, central, western, and northeastern regions under the geographical distance spatial weight matrix.

As shown by the coefficient of the spatial lag (w1*lnEF), the positive spatial spillover effect of the ecological footprint is the strongest in the eastern region, with a coefficient of 0.6346 (Table 4), significant at the 1% level, followed by the central region. This is because the development of the eastern and central regions exhibits a strong linkage effect in terms of population migration, industrial changes, and energy consumption [89]. However, because of the weak linkage effect in the western region, the spatial spillover effect of the ecological footprint is not strong, with a coefficient of only 0.3348, significant at the 10% level. In addition, although the spatial lag of the ecological footprint in the northeast is positive, it is not significant; thus, the ecological footprint in the northeastern region is not greatly affected by changes in the ecological footprint of neighboring areas. A possible reason for this result is that economic development in the northeast is predominantly based on capital-intensive industries. Enterprises with excess capacity and backward technology are too large to fail, resulting in less transfer of polluting industries. Moreover, the system is relatively rigid and degree of market freedom is low, resulting in weaker linkages in the northeast [90]; therefore, the ecological footprint of the northeastern region does not show a significant spillover effect.

Similar to the results for the whole of China, a significant inverse U-shaped relationship between innovation efficiency and the ecological footprint is found in eastern and central China. Therefore, with improved innovation efficiency, the ecological footprint of the eastern and central regions first increases then decreases. Therefore, long-term improvements in innovation efficiency will eventually significantly reduce the ecological footprint. Furthermore, the inflection point of innovation efficiency in the eastern region across the inverse U-shaped curve is 0.87. Cities that have crossed this inflection point include Beijing, Shanghai, Wuxi, Nanjing, Ningbo, Xiamen, Shenzhen, and Guangzhou. In other words, these cities have entered a stage in which the ecological footprint decreases with greater innovation efficiency. In the future, with the transformation of the economic development mode and improved innovation efficiency, some cities in the eastern region will play a leading role in improving the ecological environment and reducing the ecological footprint. It is expected that the use of various positive externalities of innovation efficiency and technological innovation will enable the comprehensive promotion and suppression of China’s ecological footprint. In the western and northeastern regions, the impact of innovation efficiency on the ecological footprint is still on the left side of the inverse U-shaped curve, indicating that greater innovation efficiency would raise the ecological footprint.

There are two main reasons for the significant regional differences in the impact of innovation efficiency on the ecological footprint in China. First, eastern and central regions have a superior technological R&D environment compared to that of the rest of the country. Moreover, with an improved innovation efficiency, scientific and technological resource inputs and technology market turnover have grown rapidly each year [91]; therefore, technological innovation has continued to improve. The degree of openness is also much higher than the national average, the market economy has a high degree of freedom, the industrial structure is continuously optimized and upgraded, and the public’s demand for improving the ecological environment is relatively high. Furthermore, as economic development differs regionally, pollution-intensive enterprises have transferred from the eastern region to the western and northeastern regions during industrialization, thereby increasing the ecological footprint in those areas. Additionally, the eastern and central regions have large populations and minimal land available for development, making them more likely to adopt a compact and intensive economic growth model that protects limited ecological resources while developing the economy. However, most cities in the western and northeastern regions still adopt the extensive economic growth model in which the positive externality of innovation efficiency on the ecological environment is not strong and the promotion of green development is relatively slow. The northeastern region, in particular, still exhibits a traditional mode of economic development characterized by high consumption, high input, and high pollution in the leading industries; thus, resource consumption continues to increase. Further, over-exploitation has caused the majority of resources to shrink to near exhaustion, which has severely damaged the ecological environment [92].

### 4.4. Economy-Related Differences in the Impact of Innovation Efficiency on the Ecological Footprint

The Hausman significance test results indicate that the null hypothesis is rejected; thus, the fixed effects model is employed for the analysis, with the ecological footprint as the explained variable and night light data (instead of economic development) as the threshold variable. The impact of urban innovation efficiency on the ecological footprint at different economic development levels is measured for the entire country and the four regions. Table 5 shows the test results. The entire country as well as the eastern and central regions passes the single-threshold test at a significance level of 1%, the western region passes the single-threshold test at 5%, and the northeastern region does not pass the single-threshold test. The national, eastern, and northeastern regions pass the double-threshold test at a significance level of 5% and the central and western regions pass it at levels of 1% and 10%, respectively. Finally, only the eastern region passes the triple-threshold test at a significant level of 10%. Because of this and the fact that the northeastern region only passed the double-threshold test, that test is used for the further analysis.

Table 6 and Table 7 show the regression results of the panel threshold model. For the entire country, when economic development is below the first threshold of 4.4928 (corresponding to night light data of 31102), the elasticity coefficient of innovation efficiency to the ecological footprint is −0.0067. When economic development is between the two thresholds, the elasticity coefficient of innovation efficiency to the ecological footprint is −0.0207. When economic development exceeds the second threshold of 4.8245, the elasticity coefficient of innovation efficiency to the ecological footprint is −0.0313. Thus, Chinese cities have achieved the win/win goal of improving innovation efficiency and reducing the ecological footprint. Moreover, the greater the urban economic development, the stronger is the reduction. Similarly, improvement in the urban innovation efficiency in the eastern and central regions has the same effect on the ecological footprint as that across the entire country. For the eastern region, when economic development exceeds the first threshold of 4.6850 and the second threshold of 4.8212, the suppression of the ecological footprint with improving innovation efficiency becomes stronger, with the coefficient changing from −0.0357 to −0.0645 (both are significant at the 1% level).

In the cities of central China, when economic development crosses the first threshold of 4.4760 and the second threshold of 4.6241, the coefficient changes from −0.0192 to −0.0365 (both are significant at the 1% level); that is, a greater innovation efficiency has an increasingly strong inhibitory effect on the ecological footprint in the eastern and central regions. In the western region, which has a higher economic development and an improved innovation efficiency, the growth in the ecological footprint is reduced and the coefficient changes from 0.0254 to 0.0192. This again illustrates that the impact of urban innovation efficiency on the ecological footprint is on the left side of the inverse U-shaped curve in western China, which is still in the transitional stage of economic transformation and development. Western regions remain the first choice for the transfer of industries with backward production capacity and environmental pollution from the east; thus, innovation efficiency does not initially have an inhibitory effect on the ecological footprint of western cities. However, only after crossing the second threshold (4.4945) does innovation efficiency have a significant promoting effect on the ecological footprint in the economically underdeveloped northeastern region (coefficient = 0.0124). Moreover, the economic development gap with other regions has continued to expand in recent years; to eliminate this gap, the focus of development in the northeast has been on increasing productivity, which neglects the effect of improved innovation efficiency on the ecological environment. Therefore, innovation efficiency does not have an inhibitory effect on the ecological footprint but does exhibit regional differences. Moreover, at different economic development levels, the impact of innovation efficiency on the ecological footprint has a double-threshold effect. Therefore, Hypothesis 2 is confirmed.

### 4.5. Impact Mechanism of Innovation Efficiency on the Ecological Footprint

An improvement in innovation efficiency may affect the ecological footprint in four ways: population aggregation, the industrial structure, the energy structure, and energy efficiency. Therefore, Equations (5)–(7) in the mediating effect model are used to test these four mechanisms. Table 8 reports the results of the mediating effect estimation. When the population aggregation effect characterized by population density is regarded as the mediating variable, the coefficients of the first and quadratic terms of innovation efficiency in Equation (7) are larger than the corresponding coefficients in Equation (5); therefore, the population agglomeration effect is not a mechanism for innovation efficiency to restrain the ecological footprint. Nevertheless, population agglomeration is still an important reason for increases in the ecological footprint. However, when the industrial structure effect is regarded as the mediating variable, the coefficients of the first and quadratic terms of innovation efficiency in Equations (5) and (6) are both significant and the corresponding coefficients in Equation (7) are less than those in Equation (5). Therefore, optimizing the industrial structure is a mediating variable for the impact of innovation efficiency on the urban ecological footprint. Regarding the energy structure effect represented by the proportion of total coal consumption to total energy consumption, the coefficients of the first and second terms of innovation efficiency in Equations (5) and (6) are both significant and the corresponding coefficients in Equation (7) are all less than those in Equation (5). Thus, the energy structure effect is also a mediating variable for the impact of innovation efficiency on the ecological footprint. Regarding energy efficiency, the corresponding coefficients in Equation (7) are less than those in Equation (5) and significant, which shows that energy efficiency is a mediating variable that affects the ecological footprint. In summary, the industrial structure, the energy structure, and energy efficiency have mediating effects on the influence of innovation efficiency on the ecological footprint. Therefore, Hypothesis 3 is confirmed. In addition, the first term of innovation efficiency is positive, whereas the quadratic term coefficients are both negative, indicating that improved innovation efficiency will eventually restrict the ecological footprint. However, if focus is placed on optimizing the industrial structure, the energy structure, and energy efficiency, ignoring the population agglomeration effect, the ability of improved innovation efficiency to reduce the ecological footprint cannot be maximized.

## 5. Conclusions

This study used panel data from 280 Chinese cities from 2014 to 2018 and the GS2SLS method to investigate the relationship between innovation efficiency and the ecological footprint. Moreover, the impact of innovation efficiency on the ecological footprint and its transmission mechanism were discussed at different economic development levels. The following three main conclusions were obtained.

First, the ecological footprint of cities across China as well as those in the eastern, central, and western regions exhibits significant spatial spillover effects, whereas that in the cities in northeastern China does not. After considering the spatial spillover effect of the ecological footprint and controlling for endogeneity, a significant inverse U−shaped relationship is observed between innovation efficiency and the ecological footprint of cities across China as well as in the eastern and central regions. That is, innovation efficiency first promotes and then inhibits the ecological footprint. However, there is no inverse U-shaped relationship between innovation efficiency and the ecological footprint in western and northeastern China, but a positive and significant relationship instead. Therefore, China should continue to adhere to the innovation-driven economic development strategy through mutual promotion and continuous improvement in innovation and economic development. Moreover, the western and northeastern regions should take the national regional economic strategy as an opportunity to integrate various innovative elements; strengthen exchanges and cooperation between scientific and technological resources, enterprises, and governments in the eastern, central, western, and northeastern regions; achieve good synergy effects; and steadily improve innovation efficiency, which will limit the growth of the ecological footprint.

Second, the impact of innovation efficiency on the ecological footprint has a double-threshold effect. Typically, with an improvement in economic development, the coefficient of the impact of innovation efficiency on the ecological footprint changes from −0.0067 to −0.0313 for the whole country, which indicates a gradual increase in the inhibitory effect of innovation efficiency on the ecological footprint. The eastern and central regions exhibit the same pattern as the entire country. However, in the western and northeastern regions, which have a greater economic development, improved innovation efficiency increases the ecological footprint, although this promoting effect has gradually weakened in the former. Therefore, China should formulate different innovation efficiency strategies according to regional economic development, actively open up the innovation chain between cities, comprehensively promote China’s innovation efficiency and technological innovation, and optimize and upgrade the industrial structure. This will gradually reduce the dependence of economic development on natural resources, promote its sustainable development, and promote greener development, thereby preventing the ecological footprint from growing further.

Finally, an improvement in innovation efficiency affects the ecological footprint through three mediating factors: the industrial structure, the energy structure, and energy efficiency. With increasing urban innovation efficiency, the population agglomeration effect does not inhibit the growth of the ecological footprint in China; instead, it increases it. Therefore, China should focus on the positive externalities of innovation efficiency to promote green technological innovation and implement relevant fiscal and tax policies to encourage and guide the development of green technology R&D activities, improve energy efficiency, reduce dependence on coal resources, and optimize the industrial structure. In addition, it is also necessary to aggressively attract high-tech industries and high-end talents, promote the joint effect of optimizing the industrial structure and energy structure and technological innovation progress, and maximize the mediating effect of innovation efficiency to reduce the ecological footprint.

## Figures and Tables

**Table 1:** Data selection and description.

Variable Type	Index Selection	Variable Name	Description	Data Source
Dependent variable	Ecological footprint	*lnEF*	Six types of land area for waste production and absorption	China Environmental Statistics Yearbook China Forestry Statistical Yearbook (2015–2019)
Core independent variable	Innovation efficiency	*lnie*	Output of scientific and technological resource input	Calculated using the SBM method
Control variable	Population	*lnpop*	Total population of a region	China City Statistical Yearbook (2015–2019)
	GDP per capita	*ln* *GDP*	GDP/population	
	Technological level	*lntec*	Total energy consumption/total energy supply	
	Household consumption	*lncosmp*	Per capita consumption of rural and urban residents	
	Pollution abatement input	*lnpolut*	Percentage of GDP invested in pollution control	
	Proportion of the secondary industry	*lnsec*	GDP share of the secondary industry	
Threshold variable	Night light data	*lnnl*	DMSP/Operational Line-Scan System night light data	NOAA website
Mediating variable	Population aggregation	*lnmidu*	Proportion of the population in the administrative area	China City Statistical Yearbook (2015–2019)
	Industrial structure	*2ndchange*	Added value of the secondary industry/GDP	
	Energy structure	*lngas*	Total coal consumption/total energy consumption	
	Energy efficiency	*lneefcy*	Total energy consumption/GDP	

**Table 2:** Benchmark regression results.

Variables	(1)	(2)	(3)	(4)
	FE	RE	FE	RE
w1*lnEF	0.7902 ***	0.9099 ***	0.7053 ***	0.7652 ***
	(0.1409)	(0.1382)	(0.2103)	(0.1800)
*Lnpop*	0.1654 ***	0.0984 **	0.1669 ***	0.0879 *
	(0.0476)	(0.0448)	(0.0475)	(0.0451)
*LnGDP*	0.3716 **	0.3486 **	0.3621 *	0.3912 **
	(0.1784)	(0.1779)	(0.1921)	(0.1915)
*Ln (GDP)^2^*	−0.0316 **	−0.2971 *	−0.0309 *	−0.0336 **
	(0.0160)	(0.0160)	(0.0171)	(0.0451)
*Lnie*	0.1654 **	0.1500 *	0.1709 **	0.1498 *
	(0.0843)	(0.0845)	(0.0839)	(0.0850)
*Ln (ie)^2^*	−0.0919 *	−0.0859 *	−0.0969 *	−0.0869 *
	(0.0665)	(0.0669)	(0.0660)	(0.0672)
*Lntec*	−0.0246 **	−0.0158	−0.0230 **	−0.0112
	(0.0120)	(0.0117)	(0.0119)	(0.0119)
*Lncosmp*			0.0792	0.0180
			(0.0660)	(0.0507)
*Lnpolut*			0.0502 ***	−0.0429 ***
			(0.0106)	(0.0106)
*Lnsec*			0.0001	0.0000
			(0.0006)	(0.0006)
C	−1.1880 **	−1.0897 **	−1.3340 **	−0.988035 *
	(0.5227)	(0.5195)	(0.6297)	(0.5802)
*Hausman test*	39.47 (0.0000)		73.85 (0.0000)	
*Adj R^2^*	0.9829	0.9829	0.9833	0.9832
*Wald test*	105.3150	92.9628	131.6106	109.0194
	(0.000)	(0.000)	(0.000)	(0.000)

Note: ***, **, and * indicate significance levels of 1%, 5%, and 10%, respectively; the values in parentheses below the coefficients are their standard errors; FE and RE indicate the fixed-effects models and random-effects models, respectively.

**Table 3:** Robustness test results.

Variables	(1)	(2)	(3)
	Replace the Spatial Weight Matrix	Replace the Instrumental Variables	Adjust the Weight Coefficient
w1*lnEF	0.7133 ***	0.6384 ***	0.5346 ***
	(0.3030)	(0.3087)	(0.1086)
*lnpop*	0.1675 ***	0.1490 ***	0.1391 ***
	(0.0473)	(0.0475)	(0.0357)
*lnGDP*	0.3691 **	0.3888 *	0.3987 *
	(0.1849)	(0.1921)	(0.1621)
*Ln (GDP)^2^*	−0.0315 *	−0.4106 *	−0.0258 *
	(0.0166)	(0.0172)	(0.1718)
*lnie*	0.1717 **	0.1662 **	0.1460 **
	(0.0838)	(0.0798)	(0.0748)
*Ln (ie)^2^*	−0.0574 **	−0.0846 *	−0.0945 *
	(0.1664)	(0.0660)	(0.4634)
*lntec*	−0.0130 *	−0.0336 **	−0.3078 **
	(0.0246)	(0.0419)	(0.0417)
*lncosmp*	0.07842	0.0662	0.0655
	(0.7456)	(0.3463)	(0.7409)
*lnpolut*	−0.0708 ***	−0.0609 ***	−0.6510 ***
	(0.0106)	(0.0213)	(0.0479)
*lnsec*	0.0000	0.0061	0.0100
	(0.0003)	(0.0006)	(0.0900)
*C*	−1.3467 **	−1.2130 *	−1.2062 *
	(0.7237)	(0.8279)	(0.6987)
*Adj R^2^*	0.9833	0.9748	0.9531
*Wald test*	131.6204 (0.000)	189.7534 (0.000)	159.6527 (0.000)

Note: ***, **, and * indicate significance levels of 1%, 5%, and 10%, respectively; the values in parentheses below the coefficients are their standard errors. In addition, owing to space limitations, this table only reports the estimated results based on the fixed effects model.

**Table 4:** Regression results for the four regions.

Variables	(1)	(2)	(3)	(4)
	East China	Central China	West China	Northeast China
w1*lnEF	0.6346 ***	0.5974 ***	0.3348 *	0.1762
	(0.2086)	(0.2028)	(0.1787)	(0.2176)
*lnpop*	0.1691 ***	0.1709 ***	0.0690	0.1526 ***
	(0.0475)	(0.0473)	(0.0446)	(0.0468)
*lnGDP*	0.3587 *	0.3773 **	0.2226 ***	0.4206 ***
	(0.1921)	(0.1851)	(0.1838)	(0.1451)
*Ln (GDP)^2^*	−0.0306 *	−0.323 *	−0.0446 ***	−0.0044 ***
	(0.0172)	(0.0166)	(0.0165)	(0.0166)
*lnie*	0.2060 **	0.1669 **	0.1278 *	0.1025 *
	(0.1038)	(0.0838)	(0.0639)	(0.0729)
*Ln (ie)^2^*	−0.0945 **	−0.0764 *	−0.0806	−0.0879
	(0.0660)	(0.0760)	(0.0694)	(0.0753)
*lntec*	−0.0510 ***	−0.0237 **	−0.0093	−0.0222 **
	(0.0106)	(0.0119)	(0.0178)	(0.0218)
*lncosmp*	0.0655	0.0595	0.0961 *	0.1045
	(0.0558)	(0.0754)	(0.0523)	(0.0652)
*lnpolut*	0.0001	0.0000	0.0013	0.0002
	(0.0006)	(0.0003)	(0.0002)	(0.0005)
*lnsec*	−0.0236 **	−0.0002	−0.1259 ***	−0.1172 ***
	(0.0119)	(0.0003)	(0.0228)	(0.233)
C	−1.2062 *	−1.1808 *	−1.3281 **	−1.3683 **
	(0.6278)	(0.6216)	(0.5688)	(0.6152)
*Hausman test*	65.44 (0.0000)	70.18 (0.0000)	73.13 (0.0000)	58.33 (0.0000)
*Adj R^2^*	0.9833	0.9833	0.9835	0.9836
*Wald test*	129.6527	129.4382	139.5025	157.9930
	(0.000)	(0.000)	(0.000)	(0.000)

Note: Owing to space limitations, this table only reports the estimated results based on the fixed effects model, and the ***, **, and * are significance levels of 1%, 5%, and 10% respectively.

**Table 5:** Threshold effect test.

Region	Entire Country	East China	Central China	West China	Northeast China
Single-threshold test	109.85 ***	45.96 ***	51.11 ***	41.37 **	21.45
	(0.0000)	(0.0067)	(0.0000)	(0.0481)	(0.2033)
Double-threshold test	79.55 **	28.04 **	33.92 ***	16.56 *	31.84 **
	(0.0233)	(0.0267)	(0.0033)	(0.0967)	(0.0578)
Triple-threshold test	23.20	29.26 *	20.28	12.66	13.53
	(1.000)	(0.0673)	(0.6133)	(0.6400)	(0.5933)

Note: The data in the table are the F-statistics corresponding to the threshold test. ***, **, and * indicate significance at the levels of 1%, 5%, and 10%, respectively, and the *p*-statistic is in parentheses.

**Table 6:** Estimates of the economic development threshold.

Model	Single-Threshold Estimate	95% Confidence Interval	Double-Threshold Estimate	95% Confidence Interval
Whole country	4.4928	(4.4904,4.4942)	4.8245	(4.8195,4.8252)
East	4.6850	(4.6790,4.6863)	4.8212	(4.8050,4.8244)
Central	4.4760	(4.4726, 4.4778)	4.6241	(4.6125, 4.6249)
West	4.4727	(4.4713, 4.4750)	4.8381	(4.8191, 4.8385)
Northeast	4.3879	(4.3853, 4.3932)	4.4945	(4.4883, 4.4996)

**Table 7:** Model parameter estimation results.

Variables	Model (1)	Model (2)	Model (3)	Model (4)	Model (5)
	Entire Country	East China	Central China	West China	Northeast China
*X (Tit < δ1)*	−0.0067 *	−0.0357 ***	−0.0192 ***	−0.0055	0.0021
	(−1.79)	(−5.98)	(−5.30)	(−0.63)	(0.38)
*X (δ1 < Tit < δ2)*	−0.0207 ***	−0.0496 ***	−0.0275 ***	0.0254 ***	0.0056
	(−7.01)	(−6.40)	(−7.75)	(3.02)	(1.18)
*X (Tit > δ2)*	−0.0313 ***	−0.0645 ***	−0.0365 ***	0.0192 ***	0.0124 ***
	(−9.69)	(−8.07)	(−8.94)	(4.04)	(2.98)
*lngdp*	0.0006	0.0011	0.0009	0.0000	0.0010
	(0.88)	(1.02)	(1.18)	(0.01)	(0.92)
*lntec*	−1.0123 ***	−0.0139 ***	−0.0022	−0.0293 ***	−0.0017
	(−5.12)	(−2.81)	(0.70)	(−5.40)	(−0.38)
*lncosmp*	0.0293 ***	0.0280 **	0.0533 ***	0.0256 **	0.0149
	(4.83)	(2.26)	(5.59)	(2.04)	(1.52)
*lnpolut*	−0.0068 ***	−0.0052	−0.0085 ***	−0.0060 **	−0.0074 **
	(−5.13)	(−1.55)	(−5.12)	(−2.22)	(−2.11)
*lnsec*	0.0001 *	0.0001	0.0001	0.0003 *	0.0002 **
	(1.84)	(0.65)	(0.94)	(1.88)	(2.19)
*C*	0.4145 ***	0.4223 ***	0.5423 ***	0.4149 ***	0.1061 *
	(11.06)	(5.78)	(9.09)	(5.23)	(1.72)

Note: t-values are in parentheses and *, **, and *** indicate significance at the levels of 10%, 5%, and 1%, respectively.

**Table 8:** Mediating effect test.

**Variables**	***D* = *lnmidu***			***D* = *2ndchange***		
	**Equation (5)**	**Equation (6)**	**Equation (7)**	**Equation (5)**	**Equation (6)**	**Equation (7)**
*lnie*	0.1176 * (0.0698)	1.62 *** (0.1102)	0.1379 ** (0.0696)	0.5460* (0.2923)	1.1855 *** (0.0697)	0.2146 *** (0.0704)
*Ln (ie)^2^*	−0.0336 ** (0.0156)	−0.1465 *** (0.0519)	−0.0379 ** (0.0152)	−0.0161 *** (0.0114)	−0.1758 *** (0.0211	−0.0133 ** (0.0175)
*D*			−0.1502 *** (0.0240)			0.1606 *** (0.0246)
**Variables**	***D* = *lngas***			***D* = *lneefcy***		
	**Equation (5)**	**Equation (6)**	**Equation (7)**	**Equation (5)**	**Equation (6)**	**Equation (7)**
*lnie*	0.1467 ** (0.713)	0.9057 *** (0.0878)	0.1271 * (0.0665)	0.1214 * (0.0701)	0.1061 *** (0.0322)	0.1141 * (0.0665)
*Ln (ie)^2^*	−0.0347 ** (0.0150)	0.0660 * (0.1139)	−0.0235 ** (0.0115)	−0.0289 * (0.0316)	−0.0620 (0.0040)	−0.0178 ** (0.0158)
*D*			0.2887 *** (0.0233)			0.1621 *** (0.0579)

Note: ***, **, and * indicate significance levels of 1%, 5%, and 10%, respectively; the values in parentheses below the coefficients are their standard errors.
